# Arousal Detection in Elderly People from Electrodermal Activity Using Musical Stimuli

**DOI:** 10.3390/s20174788

**Published:** 2020-08-25

**Authors:** Almudena Bartolomé-Tomás, Roberto Sánchez-Reolid, Alicia Fernández-Sotos, José Miguel Latorre, Antonio Fernández-Caballero

**Affiliations:** 1 Instituto de Investigación en Informática de Albacete, Universidad de Castilla-La Mancha, 02071 Albacete, Spain; almuxbt@gmail.com (A.B.-T.); roberto.sanchez@uclm.es (R.S.-R.); 2 Conservatorio de Música de Cieza “Maestro Gómez Villa”, Calle Cadenas, 6, 30530 Cieza, Spain; 3 Departamento de Sistemas Informáticos, Universidad de Castilla-La Mancha, 02071 Albacete, Spain; 4 Conservatorio de Música de Murcia, Calle Cartagena, 74, 30002 Murcia, Spain; alicia.fernandez5@murciaeduca.es; 5 Departamento de Psicología, Universidad de Castilla-La Mancha, 02071 Albacete, Spain; Jose.Latorre@uclm.es; 6 CIBERSAM (Biomedical Research Networking Centre in Mental Health), 28029 Madrid, Spain

**Keywords:** arousal, aging adults, musical genres, electrodermal activity

## Abstract

The detection of emotions is fundamental in many areas related to health and well-being. This paper presents the identification of the level of arousal in older people by monitoring their electrodermal activity (EDA) through a commercial device. The objective was to recognize arousal changes to create future therapies that help them to improve their mood, contributing to reduce possible situations of depression and anxiety. To this end, some elderly people in the region of Murcia were exposed to listening to various musical genres (flamenco, Spanish folklore, Cuban genre and rock/jazz) that they heard in their youth. Using methods based on the process of deconvolution of the EDA signal, two different studies were carried out. The first, of a purely statistical nature, was based on the search for statistically significant differences for a series of temporal, morphological, statistical and frequency features of the processed signals. It was found that Flamenco and Spanish Folklore presented the highest number of statistically significant parameters. In the second study, a wide range of classifiers was used to analyze the possible correlations between the detection of the EDA-based arousal level compared to the participants’ responses to the level of arousal subjectively felt. In this case, it was obtained that the best classifiers are support vector machines, with 87% accuracy for flamenco and 83.1% for Spanish Folklore, followed by K-nearest neighbors with 81.4% and 81.5% for Flamenco and Spanish Folklore again. These results reinforce the notion of familiarity with a musical genre on emotional induction.

## 1. Introduction

Understanding and recognizing human emotions has been identified as a main interest area in smart systems [1,2,3,4,5]. Such systems are being applied in many fields like well-being and healthcare [6,7,8,9,10,11], safe driving [12], smart cities [13] and smart environments [14,15], among others. Pleasure, arousal and dominance are three independent emotional dimensions to describe people’s state of feeling [16,17]. Arousal was conceived as a mental activity describing the state of feeling along a single dimension ranging from sleep to frantic excitement and linked to adjectives such as stimulated–relaxed, excited–calm and wide awake–sleepy to define arousal [18].

The arousal level changes constantly, and it has a profound influence on performance during everyday activities [19]. Fluctuations in arousal are regulated by the autonomic nervous system, which is mainly controlled by the balanced activity of the parasympathetic and sympathetic systems [20]. Electrodermal activity (EDA; or skin conductance) has also frequently been used as a measure of arousal. The advantage of EDA is that it is unambiguous, given that it is innervated entirely by the sympathetic nervous system (SNS) [21]. Within the domain of music emotion research, physiological measures such as EDA, heart rate, respiration, and body temperature have been frequently used as correlates of emotional arousal. Among these, EDA is generally a preferred measure as it is highly sensitive and under strict control of the sympathetic nervous system and is therefore largely involuntary. Furthermore, a relationship between EDA as indicator of emotional arousal and experienced pleasure in response to music has previously been demonstrated [22]. At the same time, in their studies with volunteers the participants’ feelings have been obtained by questionnaires in the form of Likert scales, self-assessment manikins (SAM) and free text [23,24,25,26].

This paper introduces arousal detection from EDA signals using musical stimuli. Several studies have reported that using music to elicit emotions is one of the most effective methods of emotion induction [27,28,29,30]. Music plays a key role in most people’s lives, frequently being used to explore and regulate emotions. The proposal is linked to our current research elicitation of emotions in elderly people to trigger processes of emotional self-regulation [31,32,33]. Those processes should help elderly people to improve their mood and mental state. The importance of emotional self-regulation is related to the fact that older people, especially when living alone, are at high risk of suffering from diseases such as depression and anxiety [34,35].

Specifically, people over 60 years old from the region of Murcia, Spain, were recruited as participants to listen to a series of musical pieces similar to those played in their younger years in order to study the level of arousal produced by each musical genre. Although many protocols have investigated physiological responses to music, the present work explores the physiological responses to pieces of music composed specifically for this experiment. The use of original pieces of music, which had not been heard by the listener before, is a novel research technique that has yielded interesting results so far [27,28,29,30]. The use of this type of music fragments provides a high level of experimental control and allows knowledge of the influence of the independent variables on the dependent ones. Experimental control is especially important when analyzing physiological responses like EDA [36]. The signals collected during the experiment were used in conjunction with a SAM questionnaire to undergo a couple of studies oriented towards discriminating the arousal. One study analyzed some EDA features only, and the second, based on classifiers, examined possible correlations between the objective detection of the arousal level from processed physiological EDA signals and the level of arousal subjectively perceived by participants when answering the SAM questionnaire.

The remainder of the paper is as follows. Section 2 shows the materials and methods needed to perform the experiment successfully, as well as the investigation methods and metrics used. In Section 3, the results obtained are shown and a discussion about the results obtained in the context of the experiment is provided. Finally, in Section 4 the results obtained in this study are presented.

## 2. Materials and Methods

This section describes the methodology and materials required to carry out the proposed experiment. First, an introduction is made about the electrodermal activity as a biomarker of activation detection. Then, a description of the material used, and the processes required to detect the level of activation is made. Next, the methods of data collection (SAM questionnaires) and how they are used within the experiment are explained. Afterwards, a detailed explanation of the experiment is given. Finally, the process of data segmentation and feature extraction for further analysis is explained.

### 2.1. Electrodermal Activity

Electrodermal activity (EDA) reflects the output of the attentional and affective and motivational processes integrated within the central nervous system that act on the body [37]. When emotional arousal increases, the accompanying activation of the SNS results in increased sweat gland activity and skin conductance. The validity of EDA as a measure of emotional arousal has been established in studies showing that EDA varies linearly with self-reported arousal when viewing emotional pictures [38]. Therefore, EDA is outstanding in behavioral medicine as a biomarker of individual characteristics of emotional response. EDA monitoring has been used for multiple applications, including assessment of anxiety and stress, detection of orientation response, providing neurofeedback for epilepsy, recognition of emotional state, and many others. In addition, EDA can be very effective in discriminating patients with depression from healthy controls [39]. Specific patterns of electrodermal hypoactivity may be a reliable marker of a depressive state at population level, but they should be carefully combined with other physiological and non-physiological indicators when used for preventive and diagnostic purposes.

EDA covers the electrical variations that occur on the surface of the skin due to changes in sweat secretion. EDA signals are obtained by measuring the potential when a small constant current is applied between two metal electrodes (for example, chrome-silver electrodes). The skin usually responds to stress by producing an increase in sweat. Consequently, the skin’s conductivity increases. On the other hand, sweat production stops and skin conductivity is reduced when a person is subjected to a calm or neutral induction. In this study, EDA is measured at the wrist, bearing in mind that wrist biosensors are being widely adopted in conventional and commercial devices. The bracelets provide excellent surfaces for attaching the electrodes to the skin. Ideally, the proposed system should be further miniaturized to record EDA in the areas of the palm where the activity of the skin conduction response (SCR) is most pronounced, without being intrusive or interfering with daily activities.

### 2.2. Data Acquisition and Empatica E4 Device

The commercial Empatica E4 wristband has been used to carry out our experiment. The Empatica E4 bracelet is a device that allows the collection and measurement of physiological signals such as EDA, blood volume pressure, temperature and acceleration. This device has been used with good results in some previous works [14,40,41]. In this work, we have used only the EDA signals to study the possibility of determining whether significant differences occur when a participant is subjected to different musical stimuli.

An essential component of our proposal is to acquire, process and obtain a set of data that will be used for identification of the listener’s arousal. The Empatica E4 device must be firmly attached to the wrist so that the electrodes touch the skin correctly. Otherwise, if the device is not properly connected, the captured data are not valid due to manifold artefacts.

### 2.3. Participants

40 participants, all from the region of Murcia, Spain, were recruited for the experiment. These volunteers were 23 women and 17 men with an average age of 65 (SD = 6.3) and 68 (SD = 5.1), respectively. The volunteers were all in good health and cognitive conditions to perform the experiment. They were given two screening tests, the PROMIS (Patient-Reported Outcomes Measurement Information System) diagnostic test and the TYM (Test Your Memory) test for cognitive impairment. Those who scored above the cutoff point in depression and below in cognitive functioning did not participate in the study. No compensation was paid for the conduct of the study. In addition, participants were required to sign a consent form explaining the procedure and the risks that could arise from conducting the test.

The experiment had been previously validated by the Ethics Committee of the Universidad de Castilla-La Mancha in accordance with the Helsinki Declaration.

### 2.4. Self-Assessment Manikins

One way of quantifying and subsequently relating the signals obtained from EDA to each of the different musical stimuli is by using a self-assessment manikin (SAM) questionnaire [23,24]. This questionnaire is widely used in psychology to measure the subjectively felt intensity of emotions to compare with the emotional connotation of the different physiological signals captured by electrophysiological devices [42,43,44]. The questionnaire consists of a series of manikins representing different values of valence, activation and dominance [45]. In this experiment only the manikin for activation was used.

### 2.5. Music Stimuli

As mentioned above, in this experiment the key in provoking emotions is music. For this reason, eight music pieces have been specifically composed by a professional musician for this experiment. These compositions reflect some musical styles that older people listened to when they were young (more than 30 years ago). Thus, it was the first time the participants heard each of these original pieces. All eight pieces are characterized by a same main melody and eight variations according to eight musical styles. The duration of each variation was 60 s. Table 1 shows the eight selected variations of four musical genres, with each genre including two musical styles. They are “rock/jazz” (*twist* and *swing*), “Cuban” (*bolero* and *habanera*), “Spanish folklore” (*pasodoble* and *Murcian jota*) and “flamenco” (*fandango* and *petenera*), respectively.

The musical genres used in this experiment and their repercussion in the region of Murcia are briefly described below. First, flamenco, which has been widely disseminated on the radio and orally through simple songs, is a deeply rooted genre in Spain. The most cheerful, folkloric and festive flamenco styles were adopted, such as the *Fandango* and the *Petenera*, relegating everything related to the “jondo” singing to a secondary position [46]. Secondly, Spanish Folklore, mainly linked to moments of celebration, is characterized by its joyful and jovial character. Also profoundly anchored in the popular, its simplicity and the repetition of melodic-rhythmic elements give off energy and vitality. It is closely linked to dancing as a couple, allowing one to enjoy the social atmosphere and to relate the music to the parties and the cortege.

On its side, Cuban music evokes silent listening without movement or slow dancing in couples with direct physical contact. This musical genre has also been adopted by classical music and has been expanded mainly by the cinema and the radio due to its sentimental character. Finally, jazz and rock’n’roll imply a new way of listening and relating to music. The orchestration of this music that adds instruments and sounds unknown in their culture was novel to the participants. This music relies on simple and repetitive structures, as well as on melodic improvisation through instrumental or vocal solos. The dancing of this music is also new, in pairs but without physical contact, and with very rhythmic movements that sometimes are perceived as transgression.

### 2.6. Experimental Design

An appropriate experimental design is fundamental to achieving relevant results. The E-Prime software has been chosen to create the basic design of the experiment. This software is the most widely used in the field of psychology for setting up experimental trials. In fact, E-Prime is a very robust software tool for our proper study, since it allows us to randomize and synchronize the musical pieces that are played to the participants. Furthermore, it makes it possible to add the SAM questionnaire and to control/record different parameters that will be used the exploit the EDA signals acquired during music performance.

**Figure 1:**
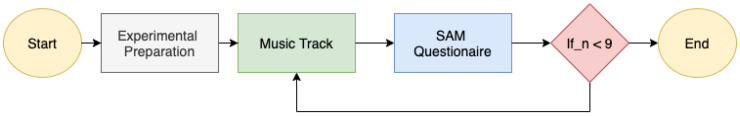
Flowchart of the experimental design

The design of the experiment has been carried out following the scheme shown in Figure 1. As it can be seen, the experiment has well-differentiated phases. In the first phase, the measuring instruments are placed on the participant. The EDA signals start to be collected when the participant is prepared, which means that he/she is in a neutral emotional state. To achieve this state, the participant remains silent looking at a black screen before the first piece of music is played. In the second phase, the participant listens to each of the musical pieces and, when the reproduction of each one of them is concluded, the person completes the SAM questionnaire. This process is carried out 8 times until all the musical pieces have been played.

At the same time as the experiment was being conducted, the EDA signals were continuously collected, making possible further segmentation, preprocessing and analysis of the signal.

### 2.7. Electrodermal Activity Preprocessing

As discussed above, EDA has been measured by a non-invasive device. Concretely, the E4 Empatica bracelet measures the skin conductance (SC) in the form of EDA signals. These measurements are composed of two signals: a first signal that varies slowly, called the tonic driver or skin conductance level (SCL), and the second that varies rapidly, called the phase driver or skin conductance response (SCR). The SCL signal establishes the base level of the signal, while the SCR is directly associated with the activity of the sweat motor system which, in turn, is directly associated with the parasympathetic nervous system.

Within the process of processing the EDA signals, different phases are crossed during which the signals are transformed. These phases are usually preprocessing, filtering, artefact removal and discrete deconvolution. The preprocessing process is in charge of establishing the segments acquired in each of the phases of the experiment. Then, it is necessary to filter the SC signals to eliminate the artefacts and interference recorded during the acquisition phase. In our case, two different filters have been used: first, a low-pass filter with a 4 Hz cutoff frequency, and second, a Gaussian filter to smooth the signal and attenuate artefacts and noise.

**Figure 2:**
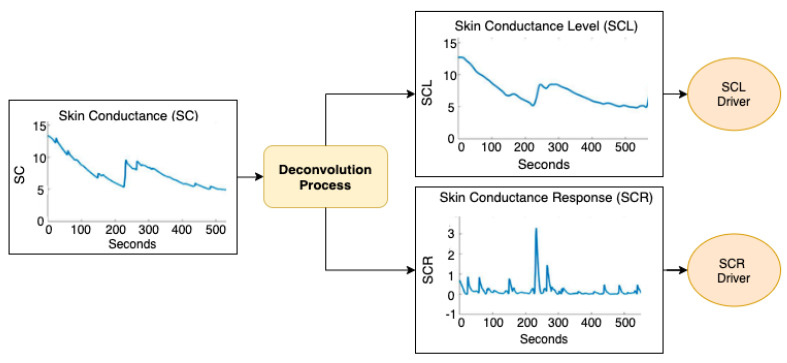
Flowchart of the deconvolution process

The next step is the deconvolution process to separate the SCR from the SCL signals. This method makes it possible to minimize the effects that race, sex and age contribute to the SC signal. Figure 2 shows an outline of how this process has been performed. As can be seen, it is the SCR driver that can be used to detect the arousal level of the participant. For this sake, the MATLAB library called Ledalab 3.4.9 has been successfully used [47]. Mathematically, the sudomotor nerve activity can be considered a *Driver* containing a train of impulses that develop over time. This response is integrated in SC and, consequently also in SCR and SCL. The result is represented by a convolution (*) of the driver with the impulse-response function (IRF), which describes the flow of the impulse response over time, as shown in Equation (1).

(1) $$SC=SC_{Driver}*IRF$$

The SC signal is composed of signals SCL and SCR, as shown in Equation (2).

(2) SC=SCL+SCR

(3) $$SC=(SCL_{Driver}+SCR_{Driver})*IRF$$

Thus, by deconvolution of Equation (3), the tonic signal driver is obtained as:

(4) $$SCR_{Driver}=\frac{SC}{IRF}-SCL_{Driver}$$

At this point the resulting signals can be used in the following process, which is feature extraction and analysis.

### 2.8. Feature Extraction and Analysis

**Figure 3:**
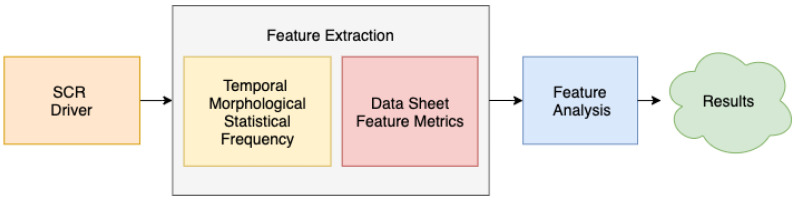
Flowchart of the feature extraction process

As commented above, to establish if there are differences between the EDA signals produced during the listening to the different music tracks, the $SCR_{Driver}$ has been used. Figure 3 shows the feature extraction and analysis process, which aim is to assess those features (metrics) that characterize the signals. The SCR driver, obtained through the deconvolution process described above, is decomposed into a series of temporal, morphological, statistical and frequency features. These features are stored on a feature sheet for later analysis to investigate if there are differences in the arousal on the basis of each feature for each of the musical genres.

Notice that the human reaction against a specific stimulus is usually expressed as a peak or a burst of peaks in $SCR_{Driver}$ as per the level of alertness involved. From a physiological perspective, the reactions against the stimuli are plotted on the signals as peaks proportional to the intensity, length and number of emotional events. The greater the disturbance caused, the greater the peak height produced in the SCR data. The number of peaks in $SCR_{Driver}$ increase when the stimulus is maintained over time, which produces a series of sequential peaks.

Table 2 details the several features selected to characterize the different segments of the $SCR_{Driver}$. These features, which have been applied successfully in previous works [40,41,48], allow us to quantify each signal.

The temporal parameters are the mean value (M), standard deviation (SD), maximum and minimum peak value (MA and MI), and dynamic range (DR) establishing the difference between maximum and minimum. These parameters can provide globally significant feedback about the average and variability of the data series. They provide specific information about a higher or lower reaction obtained through the data, which may differ by the nature of the stimulus. Other temporal parameters used are the first and second derivative (D1, D2), their means (D1M, D2M) and their standard deviations (D1SD and D2SD). The use of these parameters is due to the fact that if the stimulus is intense it produces a greater slope than when it is less intense. It is, therefore, necessary to establish a criterion of speed and acceleration in the response. If the slope has reached its maximum, the time needed in the recovery produces a smoother and opposite sign gradient.

Within the morphological features there is arc length (AL), integral area (IN), normalized mean power (AP), root mean square (RMS), perimeter and area ratio (IL), and energy and perimeter ratio (EL). These parameters obey the need to understand the morphological differences in the shape of the $SCR_{Driver}$. There are not only peaks to be studied, but changes in the general morphology of the signals are of interest. Statistical features employed are skewness (SK), kurtosis (KU) and momentum (MO). These supply information about the distribution and variability of the data series. Finally, for the frequency domain the fast Fourier transform (FFT) for bandwidths F1 (0.1, 0.2), F2 (0.2, 0.3) and F3 (0.3, 0.4) has been chosen. Using these parameters enables discovering any variation in the frequency domain for each of the stimuli.

## 3. Results and Discussion

This section presents the results obtained in the experiment, broken down into two different studies. In the first study, a series of statistical tests were carried out to determine whether any significant statistical differences exist for each the temporal, morphological, statistical and frequency features described above in the EDA signals processed for each of the music genres. The objective was to identify the variations in arousal depending on the music genre, as well as to specify which features can confirm a significant statistical difference.

The second study consisted of analyzing whether there is a clear correspondence between the responses given by the participants in the SAM activation questionnaire and the physiological EDA signals acquired during listening to the music fragments. To this end, objective information on each of the EDA signal segments associated with each music genre was linked to the subjective response to the SAM questionnaire. Several classifiers were used to quantify whether there are differences between low and high excitation states. Our purpose was to check whether these classifiers can classify the states with good accuracy.

For the statistical analysis of both studies IBM SPSS Statistics version 23 was used. Please note that in all cases only a *p*-value < 0.05 was considered to be statistically significant.

### 3.1. Direct Arousal Detection from Electrodermal Activity

As mentioned before, first a statistical study was carried out to determine if there are any significant statistical differences for each of the features selected. This started by verifying whether the features obtained from the SCL driver signals satisfied the hypothesis of normality. This check defines whether a parametric or non-parametric test can be used. In our case, all the features were found to meet this criterion with a *p*-value < 0.05. Therefore, we chose to use the T-Student distribution to determine whether significant statistical differences existed. For each of the musical genres, the comparison was made with the values obtained at the beginning of the experiment, corresponding to each participant’s neutral state (no music played). Table 3 shows the mean and the standard deviation of each of the features associated with the different musical genres. Hence, the *p*-value of each feature is provided for every musical genre in Table 4.

**Figure 4:**
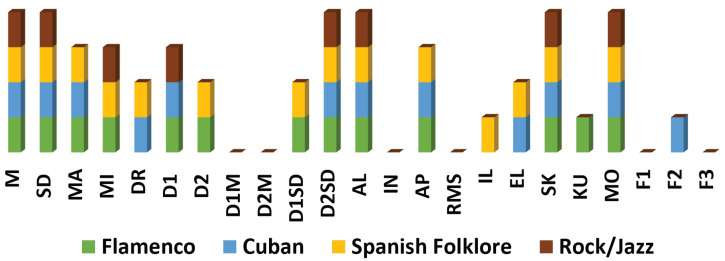
Statistically significant features for each of the musical genres according to their *p*-value.

Moreover, Figure 4 visually displays the statistically significant features for each of the musical genres. From the previous figures and table, it can be observed that the musical genres with more statistically significant differences, according to the features employed, are Flamenco and Spanish Folklore. In contrast, there are far fewer statistically significant differences in Cuban and Rock/Jazz genres.

In relation to the temporal features, M, SD and D2SD show significant differences for all four musical genres. Most other features also obtain statistically significant differences in two or three musical genres. Only for D1M and D2M there is no statistical evidence of a difference. For the group of morphological features there are only meaningful differences for all four musical genres in AL. AP presents meaningful differences in flamenco, Cuban and Spanish folklore. AP presents significant differences in flamenco, Cuban and Spanish folklore, followed by EL which has only Cuban and Spanish folk. For RM and IL no remarkable differences are found. Regarding statistical features, there are significant differences for all musical genres in SK and MO. On the contrary, for KU there are only differences in flamenco. Finally, in the category of frequency parameters, only F2 presents significant differences.

A plausible interpretation to the fact that more statistically significant differences are found in Flamenco and Spanish Folklore in contrast to Cuban and Rock/Jazz genres is provided next. Especially in the south of Spain, including the region of Murcia, flamenco is a genre that was strongly interpreted in the 60s and 70s, both in social life and in learning moments. We can say that there are many orally transmitted songs with a flamenco influence in the Spanish culture that over decades, have been sung and clapped in groups. Moreover, flamenco became a sign of identity of the purely Spanish [49]. On the other hand, through Spanish folklore, the choirs and dances, understood not as isolated elements of each Spanish region, but through musical bases common to the whole Spanish territory, were used for decades to strengthen the idea of unity of the homeland [50]. Moreover, the *Pasodoble* style and especially the *Murcian jota*, as its name indicates, are profoundly established in the region of Murcia.

On the other hand, in the 60s and 70s, and even earlier, foreign music, especially American music, was identified as the antithesis of Spanish music and as contrary to Spanish values and morality [51]. This led to the discrediting of these musical genres by the radio and the press. This was the case, although not to a high degree, of the Cuban genre. Finally, despite the media pressure of aversion towards foreign music, and mainly in foreign languages, there was an increase in fans of musical genres imported from the United States in the two great Spanish cities, Madrid and Barcelona. In small cities more rooted in traditional culture, such as the region of Murcia, these cultural manifestations had to wait a longer time [51].

### 3.2. Comparison of Arousal Detection and SAM Questionnaire Responses

The second study introduced the use of classifiers to verify that the differences between the two states (low and high arousal) mentioned above do exist. The classifiers were required to analyze possible correlations between the objective detection of the arousal level from processed physiological EDA signals and the level of arousal subjectively perceived by participants when answering the SAM questionnaire.

It was decided to use different well-known classifiers, which were grouped into trees, ensemble, regression, discriminant, naïve Bayes, k-nearest neighbors (KNN) and support vector machines (SVM). In addition, several standard configurations were chosen [52,53,54,55,56]. More concretely, we used logistic regression and linear discriminant classifier. We tried with both Gaussian and Bayes distributions in the case of naïve Bayes. Three were the configurations used for trees, namely fine tree (Gini criterion and 4 splits), medium tree (Gini criterion and 20 splits) and coarse tree (Gini criterion and 100 splits). The kinks of ensemble trees were boosted, bagged, RUS boosted and subspace KNN. The KNN configurations used were fine (Euclidean distance and 2 neighbors), medium (Euclidean distance and 10 neighbors), coarse (Euclidean distance and 100 neighbors), cosine (angular distance and 10 neighbors) and weighted (Manhattan distance and 10 neighbors). Lastly for SVM the following configurations were studied: linear (polynomial kernel, grade 1), quadratic (polynomial kernel, grade 2), cubic (polynomial kernel, grade 3) and linear (radial basis function kernel), all of them with $10^{5}$ iterations and MSE criterion.

As input parameters we used the different established features. As output we used the answers to the SAM excitation questionnaires completed during the experiment. Thirty iterations were performed for each of the classifiers, obtaining the precision (and its standard deviation) shown in Table 5. The dataset was randomly separated into 70% for training, 15% for testing and 15% for validation.

As a result, it can be seen that in the tree classifiers, for the Flamenco and Spanish Folklore genres, the tree that best classifies is the medium one with 75 and 78% respectively. On the contrary, for the rock/jazz genre, none of the trees exceed 50%, so we cannot consider that it is classified well enough. In the logistic regression classifier the results are between 60 and 67% for all music genres. One could argue that this is not a good classifier for this data set. For the linear discriminant, it was found that the best result obtained was for flamenco with 57%, which was not enough to accept it as a good classifier. Thus, this method of classification can be discarded. This is because this type of classifier works better with time series, as opposed to our proposal which is for the chosen features [57].

Naïve Naive Bayes only works well for the Gaussian configuration with an accuracy of 70.6%, 71.1% and 70.6% for flamenco, Cuban and Spanish folklore, respectively, and slightly worse for rock/jazz with 69.2%. The results of the above classifiers are in line with other studies carried out in recent years [58,59]. As for the ensemble trees, the configuration that performs the best classification is the subspace KNN. It classifies quite well the high versus low arousal states for the flamenco, Cuban genre and Spanish folklore with 74.5%, 71.43% and 72.1%, respectively. For rock/jazz the one that works better is the RUS boosted with 68.6% accuracy. The results are similar to those found in recent previous studies with EDA [60,61].

Among the KNN methods, the best classifier for flamenco is cosine KNN with an accuracy of 81.4%. For the remaining musical genres, the best is the medium configuration with an accuracy of 80.2, 81.5 and 76.09% for the Cuban, Spanish folklore and rock/jazz genres, respectively. Finally, for SVM the best classifier is the radial basis function kernel with 87.4, 81.4 and 83.1% accuracy for flamenco, Cuban genre and Spanish folklore, respectively. On the other hand, in the rock/jazz genre, the accuracy of the classifier increases to 67.4%, but it is not enough to conclude that it classifies well between the two states (low and high arousal) [62,63,64].

As is known from previous preliminary studies [40], kernel-based classifiers (SVM) perform better than the others because they can handle a larger number of features. Afterwards, distance-based classifiers of the k-NN type are the best for classifying this type of signals as may be seen from the results (see Table 5).

## 4. Conclusions

In this paper, we have presented a solution for the detection of the level of arousal from electrodermal signals (EDA) in people through their exposure to musical stimuli. For this purpose, participants over 60 years old from the region of Murcia, Spain, were recruited to listen to a series of musical pieces similar to those performed in their youth. During the playback of the music, the EDA of the participants was continuously monitored. The EDA signals acquired during the experiment were then used, along with a SAM questionnaire filled out by the participants, to conduct a couple of studies. A first study looked at the features of EDA and their ability to check for statistically significant differences for each feature extracted. A second study used well-known classifiers to analyze the potential correlation between the objective detection of the level of excitation of processed physiological EDA signals and the level of arousal subjectively perceived by participants when answering the SAM questionnaire.

The first study was based on the analysis of the existence of some kind of statistically significant difference in the selected features. The study found a greater number of statistically significant differences in the musical genres of Flamenco and Spanish Folklore, and much less in the genre of rock/jazz, which seems reasonable in the Spanish region under consideration. One of the most important factors determining musical preferences is familiarity. In accordance with our study, becoming familiar with a particular piece of music has demonstrated to increase a subject’s level of enjoyment [65,66,67,68]. This is true for emotional and autobiographical memory experiences provoked by musical stimuli [68,69]. The use of new musical stimuli allows us to control familiarity, since they are stimuli that have not been heard before. In this work the use of the same neutral melodic base in all the musical fragments (own design of the study) on different musical styles was considered. The only variation in the experiment are the musical genres, so any differences we may find must be due to the styles and not to familiarity with the musical stimulus. Considering that EDA is very sensitive to familiarity and prior exposure, the use of the procedure used in this proposal provides an important advance in music psychology research.

The second study, based on classifiers, provided information on the ability to distinguish between low and high arousal levels using both the processed EDA signals and the responses to the SAM questionnaire completed by the participants. This second study concluded that SVM, KNN and ensemble trees are classifiers that work very well in this case. Other classifiers such as linear discriminant and logistic regression did not work well for any music genre. In relation to the second study, this work has some limitations both in terms of the number of participants and the selection of the EDA signal features. In first place, a larger number of participants would be necessary to reinforce the results obtained in this study. Second, despite the large number of features used during the machine learning process, overfitting was not detected in the experiment presented. Nonetheless, a more in-depth investigation on the reduction of the features would be of interest.

This study has another limitation that has to do with the evaluation of the participants’ musical experience. Although stylistic variations of a new piece have been used, the previous exposure to the styles may not have been the same for the participants. Therefore, a system should be developed to evaluate the baseline of each participant in future studies.

The main contribution of this article has been the study of different music genres in older people to achieve a positive influence on their emotions and thus mitigate negative effects such as anxiety and depression. That contribution is based on the possibility of raising the arousal produced by memories evoked from their youth through the music heard at that time. The results of this work open the door to further studies on the fluctuations of EDA in older people with depression and/or cognitive impairment. We believe that these discoveries are expandable by developing new automated systems to help older people in their daily lives. Based on the results achieved in this experiment, we will be able to develop ambient intelligence systems to improve the quality of life and well-being of the elderly.

## Figures and Tables

**Table 1:** Musical genres and styles used in the experiment.

Musical Genre	Style
**Rock/Jazz**	*Twist* and *Swing*
**Cuban**	*Bolero* and *Habanera*
**Spanish Folklore**	*Pasodoble* and *Murcian jota*
**Flamenco**	*Fandango* and *Petenera*

**Table 2:** Features obtained from skin conductance response (SCR)

Analysis	Features
**Temporal**	M, SD, MA, MI, DR, D1, D2, D1M, D2M, D1SD, D2SD
**Morphological**	AL, IN, AP, RMS, IL, EL
**Statistical**	SK, KU, MO
**Frequency**	F1, F2, F3

**Table 3:** Mean and standard deviation for the different features.

Type	Feature	Neutral	Flamenco	Cuban	Spanish Folklore	Rock/Jazz
Temp.	**M**	5.53 (4.00)	10.01 (6.62)	8.34 (6.60)	13.01 (8.62)	7.34 (1.80)
	**SD**	4.52 (4.76)	6.34 (2.79)	6.10 (1.70)	8.69 (5.50)	6.42 (3.40)
	**MA**	28.43 (26.67)	33.32 (6.23)	34.32 (5.20)	37.10 (1.82)	35.51 (4.24)
	**MI**	0.51 (0.13)	0.81 (0.60)	0.64 (0.51)	0.66 (0.38)	0.66 (0.38)
	**DR**	28.43 (6.67)	29.79 (6.21)	34.83 (17.21)	25.83 (2.69)	29.82 (6.43)
	**D1**	0.98 (0.14)	1.13 (0.45)	1.07 (0.23)	1.09 (0.28)	1.03 (0.07)
	**D2**	0.56 (0.20)	0.71 (0.38)	0.67 (0.51)	0.86 (0.54)	0.71 (0.54)
	**D1M**	0.86 (0.13)	0.90 (0.12)	0.74 (0.44)	0.93 (0.59)	0.74 (0.44)
	**D2M**	0.45 (0.17)	0.426 (0.02)	0.48 (0.17)	0.52 (0.26)	0.55 (0.31)
	**D1SD**	0.99 (0.96)	1.23 (0.36)	1.43 (0.29)	1.40 (0.21)	1.40 (0.21)
	**D2SD**	0.29 (0.01)	0.34 (0.12)	0.56 (0.39)	0.56 (0.39)	0.36 (0.12)
Morph.	**AL**	14,049.0 (99.8)	13,950.4 (388.4)	13,850.4 (606.3)	13,950.4 (348.6)	14,450.4 (890.0)
	**IN**	193.98 (148.38)	186.98 (64.76)	245.77 (86.67)	246.77 (115.67)	230.98 (75.34)
	**AP**	4.56 (9.33)	8.17 (1.97)	4.56 (9.33)	8.17 (3.12)	2.17 (2.12)
	**RMS**	7.14 (6.23)	8.96 (4.23)	10.96 (7.34)	9.80 (6.32)	8.25 (6.34)
	**IL**	5.50 (4.14)	6.32 (4.80)	5.17 (2.80)	7.43 (2.87)	6.96 (4.23)
	**EL**	0.065 (0.0013)	0.074 (0.049)	0.085 (0.054)	0.079 (0.039)	0.045 (0.099)
Stat.	**SK**	1.18 (0.98)	1.45 (0.89)	1.82(1.72)	1.69 (0.96)	1.82 (1.79)
	**KU**	1.65 (1.09)	2.67 (2.45)	1.87 (1.02)	1.89 (1.04)	1.40 (1.34)
	**MO**	2.10 (4.06)	4.21 (3.87)	3.44 (0.24)	4.01 (3.87)	3.8 (1.76)
Freq.	**F1**	2.90 (0.29)	3.60 (1.84)	3.28 (1.99)	2.78 (0.92)	3.04 (1.35)
	**F2**	0.15 (0.32)	0.20 (0.04)	0.29 (0.12)	0.24 (0.13)	0.19 (0.02)
	**F3**	0.92 (0.37)	0.79 (0.54)	0.98 (0.26)	0.98 (0.26)	1.15 (0.64)

**Table 4:** *p*-value for the different features.

Type	Features	Flamenco	Cuban	Spanish Folklore	Rock/Jazz
Temporal	**M**	**0.004**	**0.025**	**0.000**	**0.010**
	**SD**	**0.032**	**0.040**	**0.004**	**0.030**
	**MA**	**0.021**	**0.016**	**0.041**	0.116
	**MI**	**0.036**	0.120	**0.022**	**0.021**
	**DR**	0.340	**0.014**	**0.034**	0.320
	**D1**	**0.048**	**0.032**	0.260	**0.050**
	**D2**	**0.032**	0.211	**0.001**	0.116
	**D1M**	0.160	0.116	0.442	0.116
	**D2M**	0.320	0.424	0.120	0.070
	**D1SD**	**0.039**	0.074	**0.010**	0.098
	**D2SD**	**0.032**	**0.042**	**0.004**	**0.023**
Morphological	**AL**	**0.034**	**0.010**	**0.040**	**0.010**
	**IN**	1.100	0.065	0.080	0.766
	**AP**	**0.026**	**0.000**	**0.021**	0.135
	**RMS**	0.150	0.075	0.098	0.447
	**IL**	0.420	0.687	**0.012**	0.121
	**EL**	0.230	**0.021**	**0.034**	0.210
Statistical	**SK**	**0.023**	**0.045**	**0.019**	**0.051**
	**KU**	**0.018**	0.349	0.333	0.600
	**MO**	**0.013**	**0.042**	**0.034**	**0.010**
Frequential	**F1**	0.120	0.233	0.435	0.516
	**F2**	0.320	**0.011**	0.110	0.432
	**F3**	0.210	0.434	0.434	0.053

**Table 5:** Accuracy (%) of arousal assessment through different classifiers.

Classifier	Type	Flamenco	Cuban	Spanish Folklore	Rock/Jazz
Regression	Logistic	**67.0 (0.26)**	64.0 (0.19)	61.0 (1.20)	60.0 (1.01)
Discriminant	Linear	**57.0 (0.09)**	40.3 (0.03)	46.3 (0.73)	42.5 (1.47)
Naïve Bayes	Gaussian	**70.6 (0.01)**	**71.1 (0.50)**	**70.6 (0.01)**	**69.2 (0.11)**
	Standard	67.6 (0.45)	70.0 (0.00)	68.1 (0.82)	**69.2 (0.11)**
Tree	Fine	56.0 (0.12)	69.1 (0.03)	52.0 (0.02)	40.1 (0.10)
	Medium	**75.0 (0.20)**	67.1 (0.00)	**78.0 (0.06)**	45.1 (0.27)
	Coarse	70.1 (0.00)	**70.1 (0.00)**	62.1 (0.00)	52.1 (0.45)
Ensemble Tree	Boosted	72.3 (0.04)	69.7 (0.14)	76.85 (0.23)	67.3 (0.37)
	Bagged	71.0 (0.01)	67.9 (0.11)	72.0 (0.00)	68.1 (0.76)
	RUS boosted	73.0 (0.40)	70.1 (0.50)	70.9 (0.03)	**68.6 (1.20)**
	Subspace KNN	**74.5 (0.00)**	**71.43 (0.32)**	**72.1 (0.00)**	68.1 (0.20)
KNN	Fine	76.0 (0.09)	73.9 (0.10)	76.0 (0.09)	70.0 (0.00)
	Medium	82.3 (0.05)	**80.2 (0.04)**	**81.5 (0.00)**	**76.09 (1.20)**
	Coarse	80.4 (0.02)	79.1 (0.40)	77.1 (0.18)	71.09 (1.60)
	Cosine	**81.4 (0.13)**	77.1 (1.10)	77.1 (1.80)	68.18 (1.70)
	Weighted	80.9 (0.00)	79.2 (0.06)	80.9 (0.00)	75.0 (0.00)
SVM	Linear	78.0 (0.01)	73.3 (0.63)	79.1 (0.03)	**67.4 (0.60)**
	Quadratic	72.4 (0.13)	72.4 (0.13)	72.4 (0.13)	62.0 (0.13)
	Cubic	76.4 (0.60)	78.3 (0.54)	80.4 (0.53)	65.4 (0.30)
	Radial (RBF)	**87.4 (0.00)**	**81.4 (0.00)**	**83.1 (0.01)**	67.3 (0.20)

## Abbreviations

The following abbreviations are used in this manuscript:

EDA	Electrodermal Activity
IRF	Impulse Response Function
KNN	K-Nearest Neighbor
SAM	Self-Assessment Mannequin
SC	Skin Conductance
SCL	Skin Conductance level
SCR	Skin Conductance response
SVM	Support Vector Machine
